# Lexis Diagram and Illness-Death Model: Simulating Populations in Chronic Disease Epidemiology

**DOI:** 10.1371/journal.pone.0106043

**Published:** 2014-09-12

**Authors:** Ralph Brinks, Sandra Landwehr, Rebecca Fischer-Betz, Matthias Schneider, Guido Giani

**Affiliations:** 1 German Diabetes Center, Institute of Biometry and Epidemiology, Duesseldorf, Germany; 2 University Hospital, Polyclinics for Rheumatology, Duesseldorf, Germany; 3 Heinrich-Heine-University, Institute for Statistics in Medicine, Duesseldorf, Germany; Politehnica University of Bucharest, Romania

## Abstract

Chronic diseases impose a tremendous global health problem of the 21st century. Epidemiological and public health models help to gain insight into the distribution and burden of chronic diseases. Moreover, the models may help to plan appropriate interventions against risk factors. To provide accurate results, models often need to take into account three different time-scales: calendar time, age, and duration since the onset of the disease. Incidence and mortality often change with age and calendar time. In many diseases such as, for example, diabetes and dementia, the mortality of the diseased persons additionally depends on the duration of the disease. The aim of this work is to describe an algorithm and a flexible software framework for the simulation of populations moving in an illness-death model that describes the epidemiology of a chronic disease in the face of the different times-scales. We set up a discrete event simulation in continuous time involving competing risks using the freely available statistical software R. Relevant events are birth, the onset (or diagnosis) of the disease and death with or without the disease. The Lexis diagram keeps track of the different time-scales. Input data are birth rates, incidence and mortality rates, which can be given as numerical values on a grid. The algorithm manages the complex interplay between the rates and the different time-scales. As a result, for each subject in the simulated population, the algorithm provides the calendar time of birth, the age of onset of the disease (if the subject contracts the disease) and the age at death. By this means, the impact of interventions may be estimated and compared.

## Introduction

Chronic diseases impose a tremendous global health problem of the 21st century. The World Health Organization estimates that 63% of all deaths in 2008 were caused by chronic diseases [1]. Besides taking measures in politics and society, research efforts are needed to oppose this threat. In studying the characteristics of chronic diseases from a public health perspective, it is often important to consider different time-scales [2]. The age of the subjects in a population is a risk factor for many diseases. Changes in life-style and medical care influence the risk of contracting and dying from the disease on a secular scale. Thus, the incidence rate of the chronic disease as well as the mortality rates (with and without the disease) depend on the calendar time. Moreover, the mortality of people with the disease often depends on the duration since its onset. Examples are diabetes [3], [4], dementia [5], depression [6], and systemic lupus erythematosus [7]. For decision-makers all time-scales may be important.

As a hypothetical example, consider the question which health programme to choose from two possibilities A and B if the outcome of interest is the gain of life-years. Possibility A is known to decrease the incidence of the disease by 15%, and possibility B lowers the mortality of those having the disease for more than ten years by 50%. The decision depends on several factors. If, on the one hand, the incidence rate is non-zero for children only and the birth rate in the population is low, possibility A may have little effect with respect to the gain of life-years. If, on the other hand, the chronic disease has very few people reaching ten-year survival after onset, programme B can be nearly useless. In problems similar to the example, the decision-maker may face a complex interplay of epidemiological and demographical considerations.

The aim of this work is to describe an algorithm and a flexible software framework for simulation of populations moving in a multi-state model (illness-death model) that describes a chronic disease. The simulation takes into account the different time-scales: calendar time, age, and duration of the disease. Although simulations using multi-state models are subject to recent textbooks [8], to our knowledge no algorithm has been described that incorporates the effects of all the different time-scales.

## Methods

**Figure 1:**
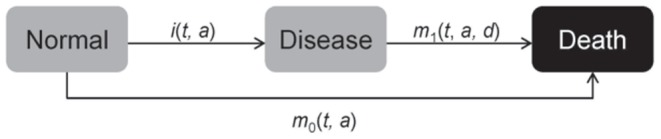
Three states model of normal (healthy), diseased and dead subjects. The transition rates may depend on calender time 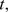 age 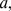 and in case of 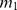 also on the duration 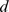 of the disease.

A popular framework for studying irreversible diseases is the illness-death model (IDM) consisting of the three states *Normal, Disease* and *Death*, [9]–[11]. The associated transition rates, synonymously *densities* (in units “per person-time”, not to be confused with risks or probabilities [12]), are the incidence 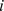, and the mortality rates 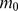 and 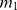 (Figure 1). In general, these rates depend on calendar time 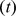, age 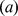 and in case of 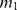 on the duration of the disease 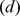.

This article presents a method for simulating populations moving in the IDM. The motivation for the algorithms comes from analytical epidemiology where relations between common epidemiological measures are studied. Examples for those measures are the prevalence, the duration of a disease, the age of onset (or diagnosis), and lost life-years (due to the disease). A typical question may be: what is the mean age of diagnosis of subjects born in a certain time period? What is their mean age at death? Another interesting aim is the estimation of the incidence rate 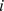 from cross-sectional information. At a specific point in time 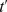, each of the subjects 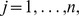 has a unique “status”. Neglecting those who are unborn or dead at 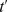, the status in the IDM is either *normal* (non-diseased) or *diseased*. Thus, the status can be seen as a binary random variable, and data of this kind are typically called *current status data* [13]. The current status is closely linked with the incidence 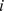 and the mortalities 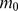 and 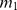 before 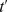. Estimating the incidence from current status data, for example, has been a topic in research for decades [14]. The framework presented here may be useful in this field.

### Overview of the simulation algorithm

The simulation is a microsimulation, i.e., it treats each person in the population as an autonomous unit. For each person, indexed 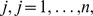 the relevant events *diagnosis* and *death* are simulated. This is accomplished in two steps:

1. Contracting the disease or dying without the disease is modelled as competing risk [11]. Given the time 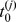 of birth of person 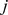, the cumulative distribution function 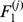 of the *first failure time* 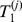 is
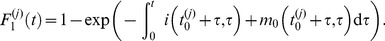
(1)The term *first failure time* 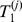 refers to the time of diagnosis or death without disease and is measured in time units after birth of person 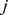. Thus, 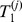 is the age at which the first transition from the state *Normal* occurs. Given that a transition occurs at 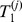 for person 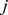, then the odds of moving into state *Disease* versus moving into state *Death* is
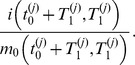

2. If the event at 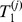 is the death (without the disease), the simulation for person 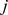 is finished. If, however, the event is the diagnosis of the disease, the “second failure time” 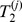 to death (with disease) has the distribution function 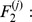


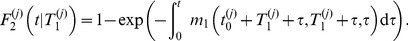
(2)

The next section describes in detail how the integrals in Equations (1) and (2) may be calculated in the simulation. After calculating the integrals, the question arises how the times 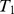 and 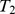 can be obtained from 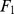 and 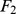. This is done by the *inverse transform sampling method*: Let 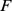 be a cumulative distribution function and 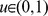. For 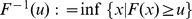 it holds: If 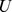 is a uniform random variable on 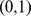, then 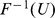 follows the distribution 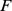. Thus, the simulation of 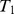 and 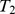 is easy, if a random number generator for 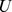 such as runif in R is available.

For each of the 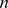 persons in the population we store four pieces of data:

1. a unique identifier 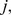

2. the date 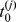 of birth (dob) of person 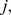

3. the age at diagnosis (adi) of person 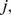 and
4. the age at death (ade) of person 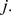

If the person 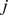 does not contract the disease, the age at diagnosis adi is set to NA (missing). In summary, we get the Algorithm 1.

**Algorithm 1** Simulation of populations moving in the IDM

1:** for** 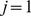 **to** 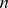 **do**

2:   dob 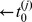


3:   calculate event time 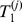 according to Equation (1)

4:   simulate type of event that has happened at 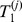 by Equation (1)

5:   **if** event is diagnosis **then**

6:     adi 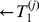


7:     calculate time 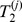 of death using Equation (2)

8:     ade 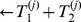


9:   **else**

10:     adi 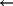 NA

11:     ade 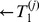


12:   **end if**

13:   write j, dob, adi, ade to file

14:** end for**

### Calculating line integrals

In this section the calculation of the integrals in Equations (1) and (2) is described. The situation in which analytical expressions for these integrals exist, is straightforward. The first simulation in the next section is an example. However, in real world applications, analytical expressions for the integrals are rarely given. For convenience, mathematical functions (e.g., splines) may be fitted to the data and integration is accomplished with the fitted functions. Since the aim of this work is a flexible way of treating the incidence and mortality rates, we assume that the rates are given as numerical values on a regular grid. Here we focus on the most general case, which is characterized by:

1. None of the time-scales 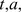 and 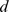 is negligible, and
2. the values of 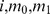 are given as data points only.

For many chronic diseases, we think this is the most relevant case: The mortality rates 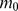 and 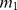 depend on 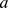 and 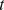. Since age is a risk factor for many diseases, the dependency on age is obvious. Healthier life-style and medical progress in many countries lead to secular trends in 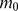 and 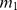. In addition, disease duration 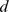 is likely to have an impact on 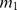 in many chronic diseases. Thus, none of the time-scales is negligible, which is covered in the second and third example.

In the most general case, the integrands 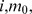 and 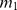 are given by data points only. We assume that the numerical values are located on a regular grid. The grid is two-dimensional in case of 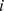 and 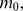 which depend on two time-scales 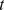 and 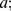 and the grid is three-dimensional in case of 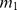 which depends on 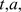 and 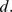


In event history analysis [2], a useful concept is the *Lexis diagram*, which is a co-ordinate system with axes calendar time *t* (abscissa) and age 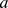 (ordinate). The *t*-dimension sometimes is referred to as period. Each subject is represented by a line segment from time and age at entry to time and age at exit. Entry and exit may be birth and death, respectively, or entry and exit in a epidemiological study or clinical trial. There are excellent and extensive introductions about the theory of Lexis diagrams (see for example [9], [15], [16] and references therein), which allows us to be short here. In irreversible diseases, the common two-dimensional Lexis diagram with axes in *t*- and *a*-direction may be generalized to a three-dimensional co-ordinate system with disease duration 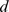 represented by the applicate (i.e., the z-axis). If a subject does not contract the disease during lifetime, the life line remains in the *t*-*a*-plane parallel to the line bisecting abscissa and ordinate. In other words, the life line for the time without disease is parallel to 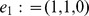 (where the triple 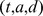 denotes the co-ordinates in time, age and duration direction, respectively). However if at a certain point in time 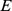 the disease is diagnosed, the life line changes its direction, henceforth runs parallel to 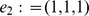.

**Figure 2:**
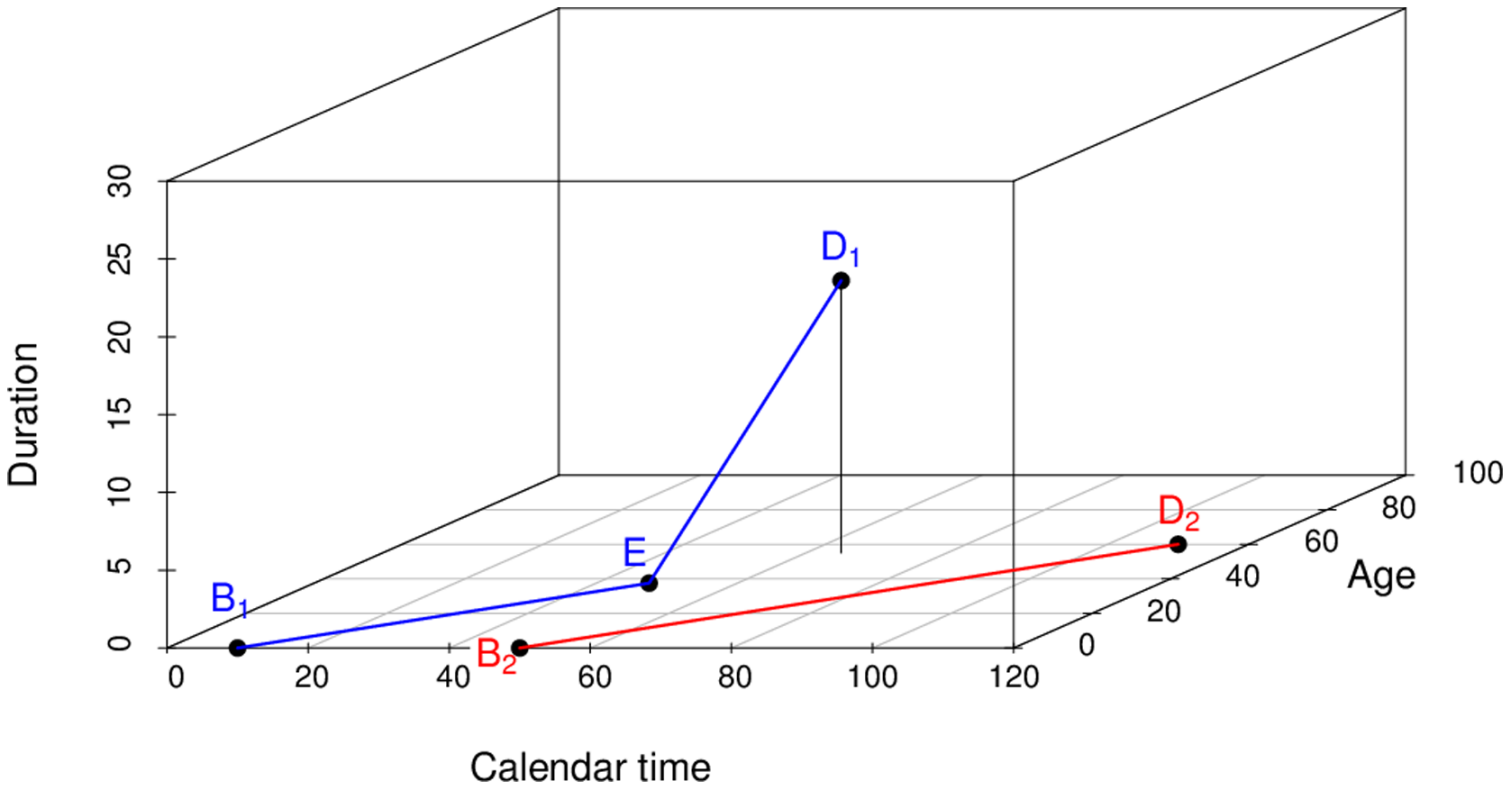
Three-dimensional Lexis diagram with two life lines. Abscissa, ordinate and applicate (z-axis) represent calendar time 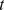, age 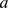 and duration 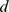, respectively. The life lines start at birth 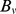 and end at death 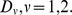 The first subject (blue line segments) contracts the disease at 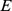. Then, the life line changes its direction. The second subject (red line segment) does not contract the disease, the life line remains in the *t*-*a*-plane.

The situation is illustrated in Figure 2. The life lines of two subjects are shown in the three-dimensional Lexis space. At birth (denoted 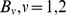) both subjects are disease-free; both life lines are parallel to 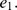 The first subject contracts the disease at 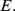 Henceforth, the life line is parallel to 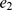 until death at 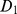. The second subject remains disease-free until death at 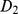.

Having the concept of the Lexis diagram at hand, we observe that 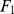 and 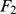 in Equations (1) and (2) are line integrals in the Lexis space. We start with calculating the first failure times 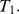 For subject 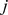 the associated life line starts at 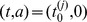. We chose an age 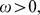 when it is certain that a transition to one of the states *Disease* or *Death* has occurred, say 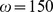 (years). For calculating 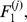 we trace the hypothetical life line from 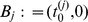 to 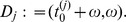 Thus, the hypothetical life line has a representation
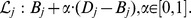



Following the life line is related to the method of ray-tracing in the field of computer graphics, where efficient algorithms for this purpose exist. In Siddon's algorithm [17], the key idea is to follow 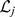 by calculating intersections with volume elements (voxels), which form a regular partition of the Lexis space. Let
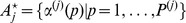
with 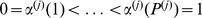 be a parametrization of the points where 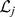 intersects the voxel faces plus the start and end points 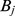 and 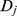. Details for the calculation of 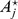 are described in the supporting information to this article. The parametrization 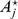 is ideally suited for approximating the integral in Equation (1) by the trapezoidal rule [18]. The reason lies in the fact that in calculating 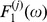 the values 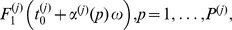 are a by-product. Algorithm 2 shows the necessary steps.

**Algorithm 2** Calculating 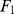


1:** for** 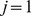 **to** 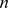 **do**

2:   calculate 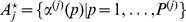


3:   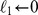


4:   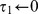


5:   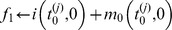


6:   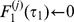


7:   **for** 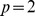 **to** 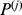 **do**

8:     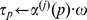


9:     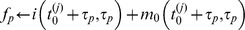


10:     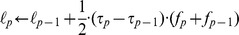


11:     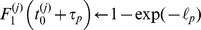


12:   **end for**

13: **end for**

Since the values of 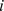 and 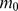 are given on the voxel grid only, the calculation of 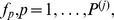 needs bilinear interpolation of the values of the adjacent voxels [19].

After preparing 
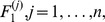
 the values of the times 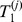 can be calculated by the inverse transform sampling method. Since we have 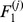 calculated at points 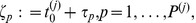 the inverse transform sampling would yield only those 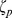. A better accuracy can be obtained by (linear) inverse interpolation [18]. For 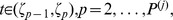 let 
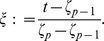
 Then, it holds
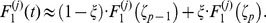



Thus, for 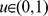 drawn from a uniform distribution, we find the unique 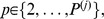 such that 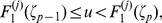 and set
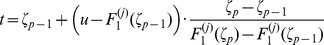
as the inverse 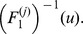


For those subjects 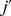 who contract the disease, the associated 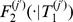 can be derived in a similar way as in Algorithm 2. The associated line segment starts at 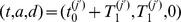. Again, a hypothetical maximal disease duration 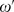 is assumed, say 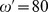 (years), such that the line segment ends at 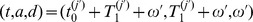. Thus, the line segment is parallel to 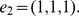 The Siddon algorithm computes the corresponding set of intersections with the voxel grid accordingly. The ages 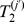 at death with disease are obtained from Algorithm 2 mutatis mutandis. The interpolation of 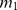 needs to be trilinear.

## Examples

This section shows the results of different simulation settings. The associated R [20] code is provided with this article. The first simulation is about a hypothetical chronic disease with rates 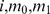 only depending on 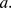 The corresponding age-specific prevalence can be calculated analytically, which allows cross-checking the results of the simulation. In the second example, another hypothetical disease is treated with mortality rates depending on 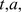 and 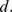 Here we use the ray-tracing approach in the Lexis diagram. Again, the outcomes of the simulation are compared with analytical results. The third simulation is about a relatively rare rheumatic disease. A hypothetical birth cohort of 100000 women is followed from birth to death and examined if the disease is diagnosed. Finally, the last simulation demonstrates applicability in the context of medical decision-making. Two interventions are compared with respect to the outcome *life-years gained*.

### Simulation 1: Analytical example

**Figure 3:**
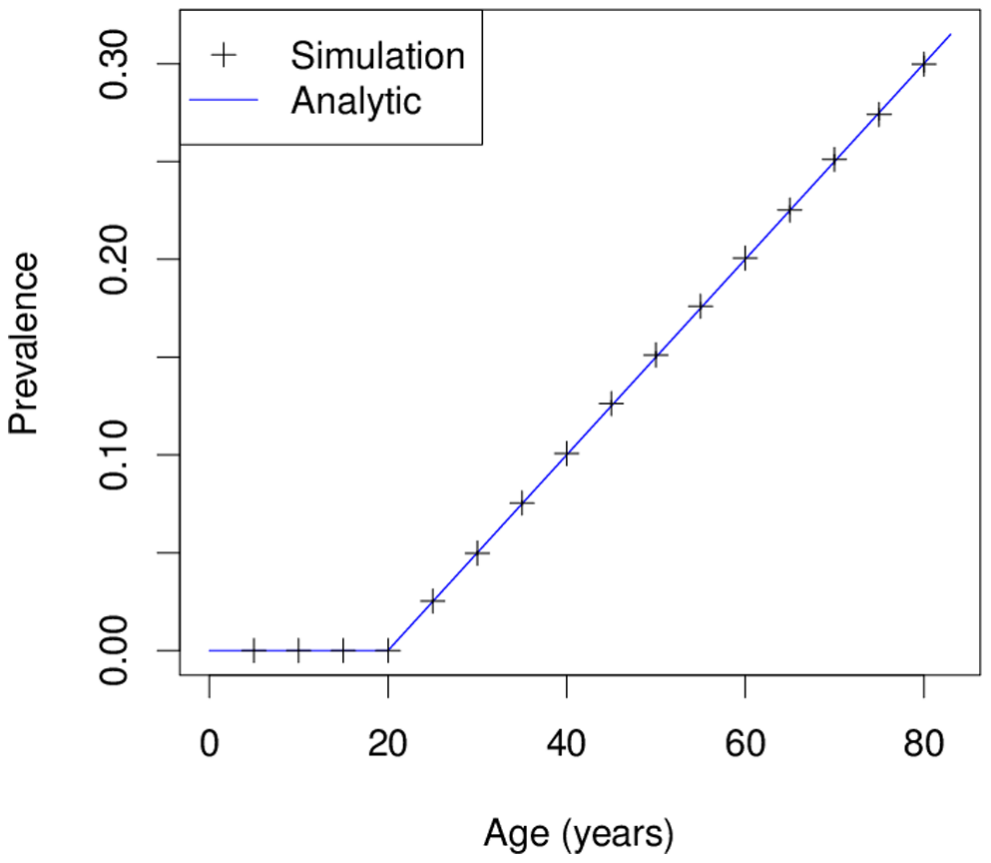
Theoretical and simulated age-specific prevalence. Simulation 1 comprises 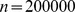 persons. The resulting age-specific prevalence (black crosses) is compared to the analytically calculated prevalence (blue solid line). The example shows the very good agreement between the simulation and the theoretical results.

In the first simulation, only one time-scale is involved. Assuming 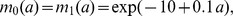 and
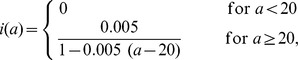
one can show that the age-specific prevalence 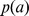 is given by 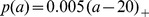 [21]. The notation 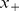 means the positive part of 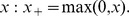 The integrals of 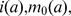 and 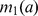 can easily be expressed analytically. Figure 3 shows the age-specific prevalence in a simulated cohort with 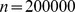 subjects. For comparison, the true (analytical) prevalence is depicted as a solid line. The result of the simulation agrees very well with the analytical age profile of the prevalence, which indicates the correctness of the implemented R code.

### Simulation 2: Mortality depends on duration

The second simulation is about a hypothetical disease with all time-scales 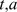 and 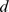 playing a role. We aim at calculating epidemiological measures that describe the population of the diseased. For example, age of onset, mean duration of the disease and age at death may be important to plan resource allocation (e.g., inpatient facilities).

**Figure 4:**
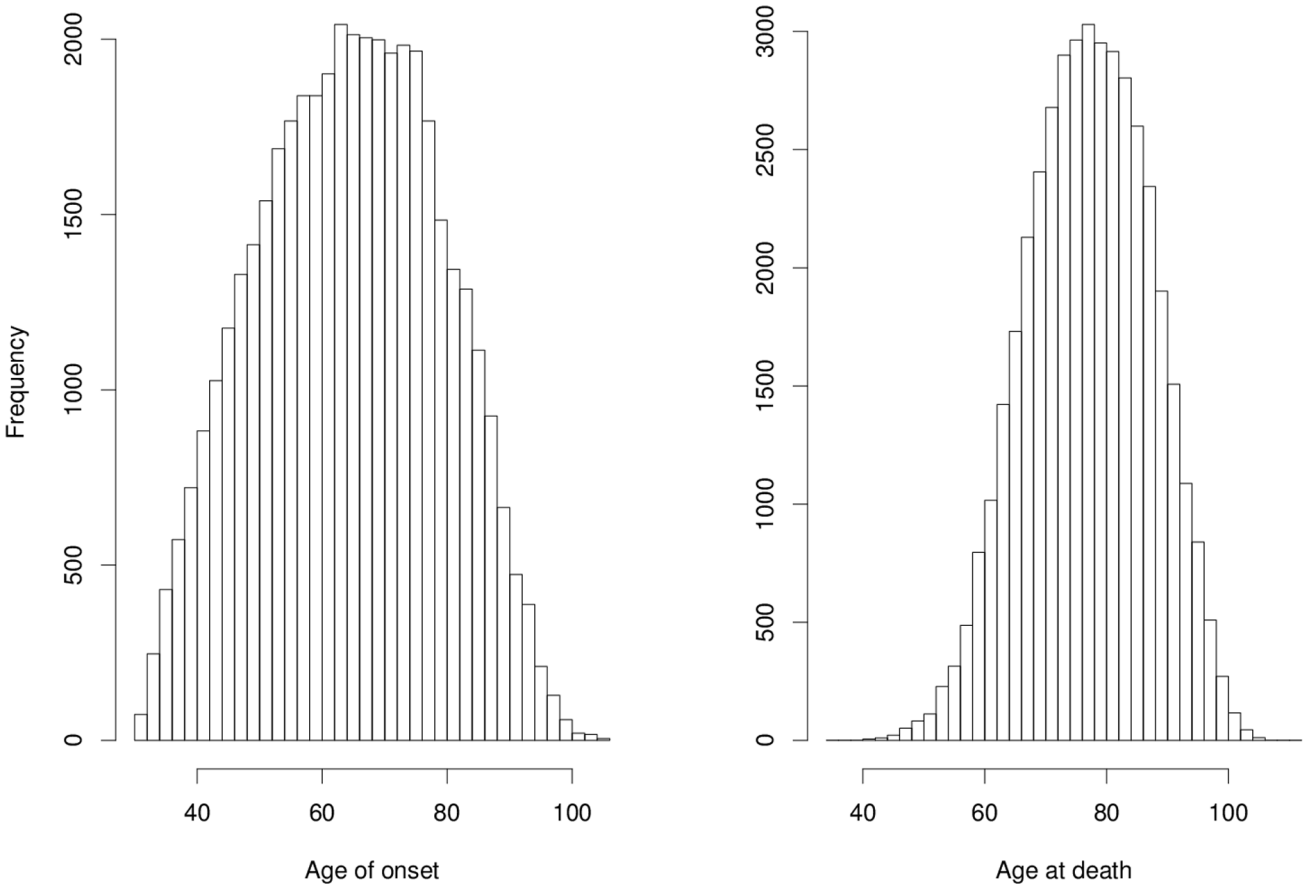
Histograms of the age of onset and age at death in a hypothetical chronic disease. In Simulation 2 the empirical distributions of the age of onset (left) and the age at death of the diseased persons (right) are estimated.

In each of sixty consecutive years 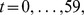 2000 persons are born and followed from birth to death. The incidence of a hypothetical chronic disease is assumed to be 
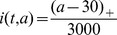
, the age-specific mortality rate of the non-diseased is chosen to be 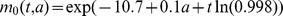 and the mortality of the diseased is 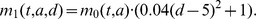 In total, 42299 of the 120000 simulated persons contract the disease. The simulated data allows the derivation of important epidemiological measures. For example, the histograms of the age of onset and age at death are shown in Figure 4.

The median age at death of those who contracted the disease is 77.20 (years) whereas the median age at death of those without the disease is 79.67 (years). The median duration of the disease in the 42299 ill subjects is 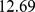, the interquartile range is 7.46–17.60 (years).

**Figure 5:**
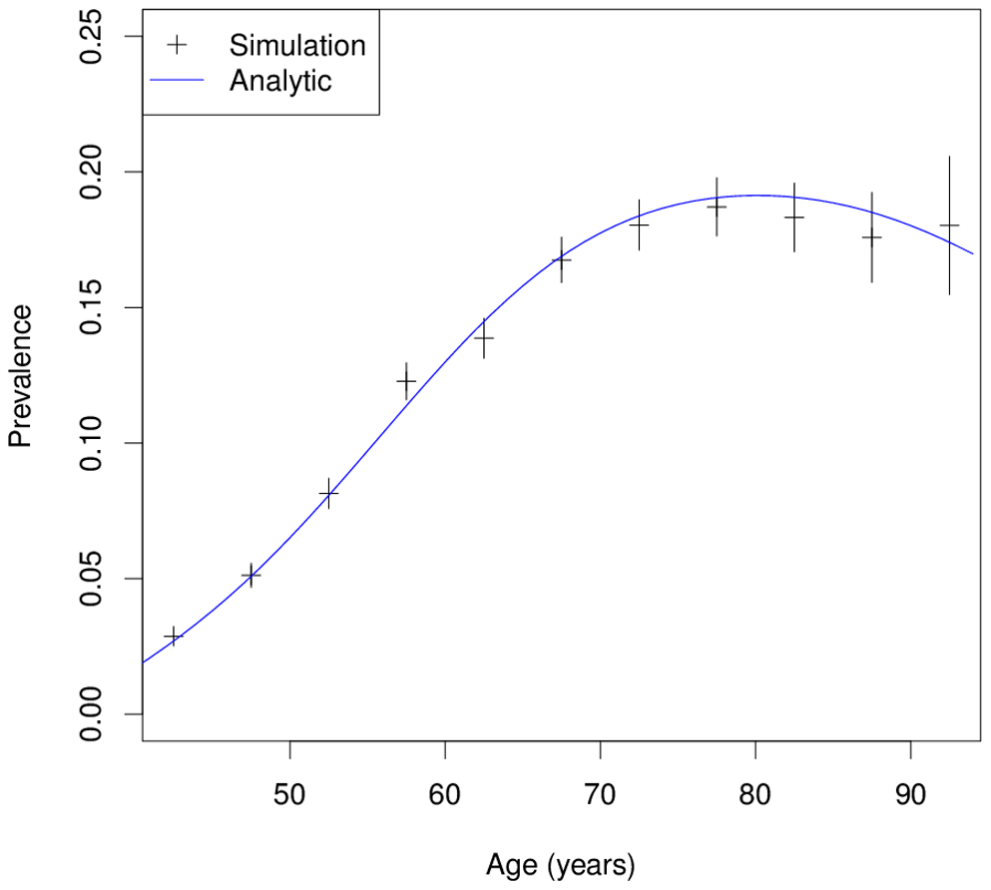
Calculated and simulated prevalence in Simulation 2. If we mimic a cross-sectional study at year 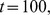 we obtain the age-specific prevalence as indicated by the black bars (with 95% confidence bounds). For comparison the analytically calculated age-specific prevalence is shown as blue line.

Finally, we can cross-check the results of the simulation by comparison with an analytical calculation. In year 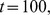 exactly 76548 persons are alive, 8802 of those having the hypothetical disease. Figure 5 shows the age-specific prevalence at 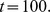 The black lines indicate the prevalence of several age groups together with 95% confidence bounds as given by the simulation. The blue line represents the prevalence calculated analytically by the exact formula in ([9], Section 7.2). The results agree quite well within the confidence bounds.

### Simulation 3: Systemic lupus erythematosus in the UK

The third simulation is about a hypothetical cohort of 100000 women born in the United Kingdom in 1945. The disease under consideration is systemic lupus erythematosus (SLE), which is an autoimmune disorder with significant morbidity and increased mortality. Women are more often affected than men; in terms of age-standardized incidence, the ratio is 5∶1 [22]. The peak of the age-specific incidence in British women is located at the age of about 50. Since Somers et al. could not find a significant secular trend in the age-standardized incidence in 1990–1999, we assume that the incidence does not depend on 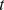, [22]. The aim of this example is to study the interplay between age of onset and the mortality of the diseased women in a realistic setting.

Mortality 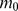 has been taken from the official life tables of the British Office for National Statistics [23]. The calendar time trend in 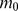 has been simplified by assuming that the yearly decrease in mortality is 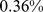 for all age groups. This is the geometric mean of the decrease in female mortality taken over all reported age groups in the past 60 years. Mortality 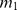 of the women with SLE has been modelled according to [7], which takes into account several covariables: sex, age, duration of SLE, calendar time of diagnosis and geographical region. Unfortunately, an interaction analysis of these factors has not been reported, which forces us to make assumptions. We assume a multiplicative model of the impact of sex, age, region and SLE duration:
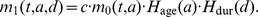



The constant 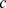 reflects the impact of sex and the region, 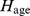 and 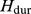 are hazard ratios. The hazard ratio 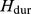 of SLE duration has been interpolated affine-linearly on a logarithmic scale from the published values and backtransformed. The age dependency of 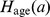 has been treated similarly. Due to the weak (and possibly insignificant) effect, the impact of the calendar time of diagnosis has been neglected.

From the 100000 women in the simulation, 513 contract SLE. This corresponds to a lifetime risk of about 0.5%. For comparison, a recent publication about women in the US estimated 0.9% [24]. The study [24] included women with African ancestors, who are known to have a higher risk. The median age of onset in the simulation is 46.07 years, which is in nearly perfect agreement with the result of 46.1 years based on another simulation using the same data but a different method [25]. Table 1 shows the interplay between age of diagnosis and duration of SLE in the simulation.

**Table 1:** Age at diagnosis and disease duration of 513 British women with SLE in a simulated cohort.

Age at		SLE duration (years)					
diagnosis		<1	1–2.4	2.5–4	5–9	10–24	≥25
5–34	(n = 134)	12.7%	9.0%	11.9%	14.9%	19.4%	32.1%
35–44	(n = 108)	15.7%	10.2%	14.8%	13.9%	19.4%	25.9%
45–59	(n = 149)	16.1%	14.1%	14.8%	13.4%	26.8%	14.8%
≥60	(n = 122)	29.5%	16.4%	21.3%	23.0%	9.0%	0.8%

Example how to read the table: 108 of the women are diagnosed in the age group 35–44, 13.9% of those die 5–9 years after diagnosis.

From Table 1 it is apparent that ten-year survival is not easy to reach. Substantial loss of life time is also indicated by the age at death: Median age at death for the diseased and the non-diseased women is 61.4 and 77.1 (years), respectively. This indicates a considerable loss of lifetime in the population of the diseased compared to the non-diseased. Fortunately, this situation has changed in the last years with better medical care for SLE patients. Especially the introduction of optimized treatment regimes in the last decade lead to an enormous reduction of mortality [26]. These effects have not been included in the simulation.

### Simulation 4: Effectiveness of two interventions

We take Simulation 2 as the basecase and compare the effectiveness of two hypothetical intervention programmes A and B with respect to the total gain of life-years. Assume that the primary prevention programme A gradually lowers the incidence rate for all 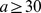 in the calendar years 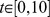 by 15% and remains at the 85% level for 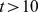. Programme B is assumed to reduce the mortality 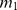 of the diseased people gradually by 50% starting after six years with the disease. B could be achieved, for example, by listing a new drug on the formulary. After running the simulation, we find that in total the 120000 simulated persons in the basecase, in intervention A, and intervention B, have 9275008, 9348941, and 9351338 life-years, respectively. Hence, intervention A and B yield about 73900 and 76300 life-years more than the basecase. With respect to the chosen outcome parameter, gain of life-years, programme B turns out to be superior to programme A. Thus, primary prevention by programme A, in this example, is less effective than lowering the mortality of the diseased. This result is hardly predictable without a simulation and demonstrates the usefulness of our algorithm in decision making.

**Figure 6:**
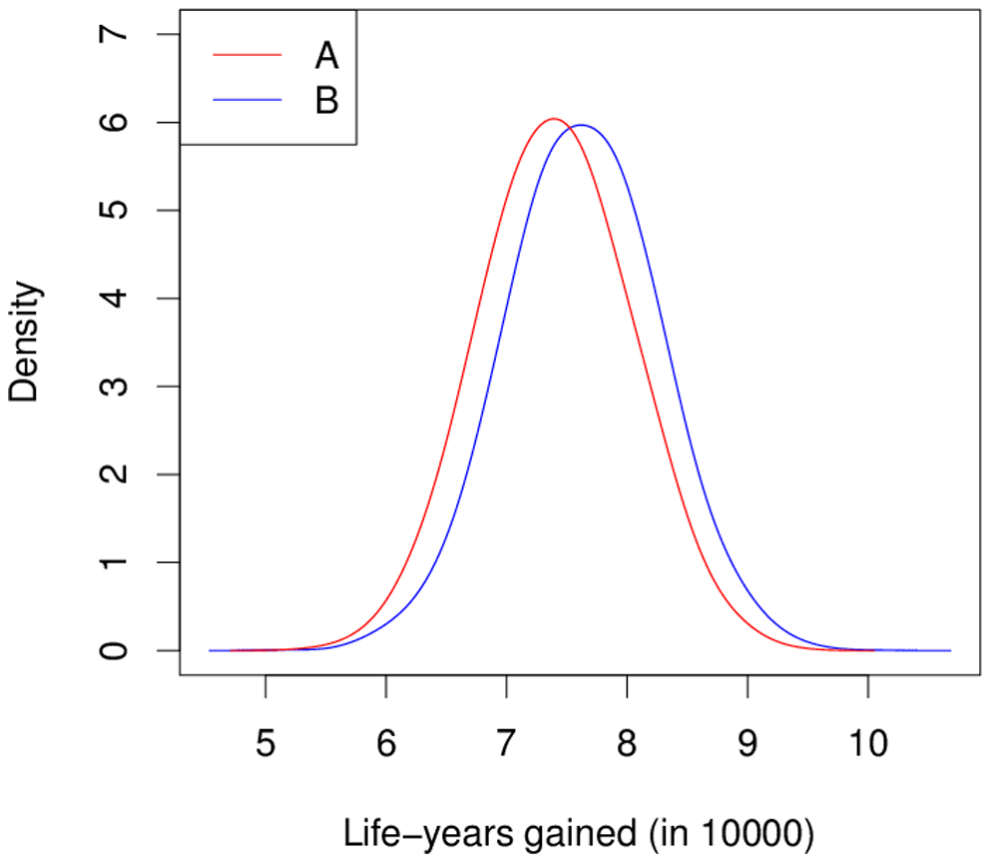
Esimtated densities of the gain in life-years in two intervention programmes. Simulation 4 compares two intervention programmes in a population of 120000 persons. The estimated densities of the total gain in life-years (compared to a basecase) are shown as red and blue curve for programmes A and B, respectively.

To obtain the empirical distributions of the total gain of life-years in both interventions, 5000 random samples of size 120000 have been drawn (with replacement from the simulated 120000 persons) [27]. Figure 6 shows the resulting densities and the superiority of programme B.

## Conclusions

This article is about simulating populations in an illness-death model consisting of the three states *Normal, Disease*, and *Death*. The relevant events, diagnosis and death, in the most general case depend on three time-scales: calendar time, age and disease duration.

After birth of a subject in the population, two cases may occur:

1. the subject dies without the disease, or
2. the subject contracts the disease and dies with the disease.

Both situations can be represented in the Lexis space, a common tool in event history analysis. In the first case, the life line is solely located in the time-age-plane. In the second case, the life line changes its direction after onset of the disease, which allows to model the duration of the disease. In many diseases, the duration plays an important role for the mortality. Beside systemic lupus erythematosus as treated above, other diseases such as diabetes [3], [4], depression [6] and dementia [5] may serve as examples.

In the most general case, the simulation requires to numerically solve line integrals. Therefore, a synthesis between a ray-tracing technique and numerical integration is exploited. The method provides a fast way to follow the individual life lines in the Lexis diagram. Computation time is an issue, because the number of simulated subjects in the population may be large (several thousands). For example, Simulation 4 takes almost nine minutes (525 seconds) on an Intel i3 personal computer with 3.3 GHz and 8 GB RAM. Simulations 1, 2, and 3 take 40, 170 and 85 seconds, respectively.

Beside the areas mentioned above, we think the method may be applicable in following the fields:

- In epidemiology, the simulation may be used to study the interplay between characteristics in chronic diseases: prevalence, mean disease duration, age of onset, age at death of the non-diseased and diseased population.
- The algorithms may serve as a test bench for estimation methods. For example, in [21] the age-specific incidence is derived from prevalence data. The simulation may be used to study the performance of this and related methods.
- In health economics, the result of our simulation allow the application of cost weights to each subject of the population. For example, in diabetes it is well-known that disease related costs depend on the duration since onset [28]. For each individual the disease related costs may be calculated at a specific point in time. By summing over all subjects, the total costs may be estimated easily.
- Similarly, for many chronic diseases, the health related quality of life depends on the duration of the disease [29]. By assigning utility weights to each individual and summing them up, the total quality-adjusted life-years (QALY) may be calculated.

The last two points are related to health economic modelling. We think that our algorithm may yield a contribution in that domain, because often health economic models are Markov models. Due to memoryless property of Markov models, the dependency of the relevant outcomes on the duration cannot be included directly ([30], Sec. 4.2.3). In the field of diabetes, for example, at least six out of ten important economic models are not capable to accurately account for diabetes duration [31]. This may not be necessary in every research question, but if highly accurate results are needed, modelling the disease duration should be considered.

## Supporting Information

File S1 (PDF)

File S2 (ZIP)
