# Supplementary material for: Lexis Diagram and Illness-Death Model: Simulating Populations in Chronic Disease Epidemiology
Source: PLoS One. 2014 Sep 12;9(9):e106043. doi: 10.1371/journal.pone.0106043 (PMC4162544; doi:10.1371/journal.pone.0106043)
Supplement: File S1 — (PDF) [file pone.0106043.s001.pdf]

Supplement to: Lexis Diagram and Illness-Death Model: Simulating Populations in Chronic Disease Epidemiology

Ralph Brinks*, Sandra Landwehr, Rebecca Fischer-Betz, Matthias Schneider, Guido Giani

* E-mail: ralph.brinks@ddz.uni-duesseldorf.de

Tracing life lines in the Lexis space

Given the life line $\mathcal{L}_j$ of subject j we want to calculate the associated line integrals in Equations (1) and (2) of the main document. The three-dimensional line integral in Equation (2) is more complex than the planar integral in Equation (1). Thus we focus on the 3D case and assume that the associated three-dimensional Lexis space is partioned into a set of regular, rectangular hexadrons (right cuboids) S_{qrs}. These congruent volume elements are called *voxels.* The six faces of each voxel are subsets of two adjacent planes parallel either to the t-a-plane, a-d-plane or t-d-plane. Hence, the voxel space comes along with a set of equidistant, parallel planes which are perpendicular to the abscissa, ordinate or applicate and which are defined by the union of all voxel faces. These planes play a crucial role in the algorithm.

In this work and the provided algorithms, all voxels S_{ijk} are considered to be cubical, with all edges having the length `timeRes` $\tau,\ \tau > 0$:

$$S_{qrs} := [\tau \cdot (q-1)\,,\ \tau \cdot q) \times [\tau \cdot (r-1)\,,\ \tau \cdot r) \times [\tau \cdot (s-1)\,,\ \tau \cdot s)\,.$$

As a consequence of cubical voxels, the temporal resolution with respect to calendar time, age and duration is the same. However, generalization to partitions using rectangular voxels with height, length and depth of the voxels being different is possible.

The main idea for tracing the life line $\mathcal{L}_j$ of subject j starting at entry point $B_j := (t_0^{(j)}, a_0^{(j)}, d_0^{(j)})$, ending at exit point $D_j := (t_1^{(j)}, a_1^{(j)}, d_1^{(j)})$, is the parameterization in the form

$$\mathcal{L}_j :\ B_j + \alpha \cdot (D_j - B_j),\ \alpha \in [0,1].$$

Note that $t_1^{(j)} - t_0^{(j)} = a_1^{(j)} - a_0^{(j)} = d_1^{(j)} - d_0^{(j)} =: \Delta t^{(j)}$. Using this parameterization, all parameters $\alpha^{(j)} \in [0,1]$ are calculated for which $\mathcal{L}_j$ intersects a voxel face. Since the voxels are arranged in a regular grid, intersecting one of the voxel faces is equivalent with intersecting one of the t-a-, a-d- or t-d-planes

formed by the union of all voxel faces mentioned above. Hence, we calculate the intersections of $\mathcal{L}_j$ with these planes.

Let us start with the a-d-planes (perpendicular to the t-axis): all those $\alpha_t^{(j)}$ where an intersection with an a-d-plane occurs are given by

$$\alpha_t^{(j)}(u) = \frac{u \cdot \tau - (t_0^{(j)} \ \% \ \tau)}{\Delta t^{(j)}}, \ u = 1, \ldots, U^{(j)},$$

where % is the modulo-operator and $U^{(j)}$ denotes the number of intersected a-d-planes:

$$U^{(j)} = \left\lfloor t_1^{(j)}/\tau \right\rfloor - \left\lfloor t_0^{(j)}/\tau \right\rfloor.$$

Similar formulas hold for those $\alpha_a^{(j)}(v)$, $v = 1, \ldots, V^{(j)}$, and $\alpha_d^{(j)}(w)$, $w = 1, \ldots, W^{(j)}$, where $\mathcal{L}_j$ intersects the t-d- or t-a-planes, respectively. Now define the set

$$\begin{aligned} A_j := \quad & \{\alpha_t^{(j)}(u) \mid u = 1, \ldots, \ U^{(j)}\} \\ \cup \quad & \{\alpha_a^{(j)}(v) \mid v = 1, \ldots, \ V^{(j)}\} \\ \cup \quad & \{\alpha_d^{(j)}(w) \mid w = 1, \ldots, \ W^{(j)}\}, \end{aligned} \tag{1}$$

which contains all those $\alpha^{(j)} \in [0, 1]$ where an intersection occurs. Note that the three sets on the right-hand side of Equation (1) are not necessarily disjoint. Multiple values occur if an intersection happens to be on an edge or vertex of a voxel. By ordering $A_j^\star := A_j \cup \{0, \ 1\}$ ascendingly $A_j^\star = \{\alpha^{(j)}(p) \mid p = 1, \ldots, P^{(j)}\}$ with $0 = \alpha^{(j)}(1) < \cdots < \alpha^{(j)}(P^{(j)}) = 1$, we are finished in sampling the life line $\mathcal{L}_j$.

The idea of calculating the intersection points with the voxel faces goes back to the seminal work of Siddon. The algorithm proposed in reference [16] of the main text has been developed for raytracing in tomography, where several millions of "life lines" (i.e., radiological paths between a light source and a detector) have to be traced to form a radiological image.
